# Genetic monitoring in ***ex situ*** populations of the endangered primate ***Leontopithecus chrysopygus*** and integrative analyses with the wild founder population

**DOI:** 10.1371/journal.pone.0322817

**Published:** 2025-05-07

**Authors:** Nathalia Bulhões Javarotti, Paola Andrea Ayala-Burbano, Alcides Pissinati, Mara Cristina Marques, Dominic Wormell, Gabriela Cabral Rezende, Laurence Culot, Pedro M. Galetti Jr, Patrícia Domingues de Freitas

**Affiliations:** 1 Departamento de Genética e Evolução, Laboratório de Biodiversidade Molecular e Conservação, Universidade Federal de São Carlos, São Carlos, SP, Brazil; 2 Grupo de Investigación Giesum, Facultad de Ciencias de la Salud, Universidad Mariana, Pasto, Nariño, Colombia; 3 Centro de Primatologia do Rio de Janeiro, Guapimirim, Rio de Janeiro, Brazil; 4 Zoológico de São Paulo, São Paulo, São Paulo, Brazil; 5 Durrell Wildlife Conservation Trust, Trinity, Channel Island, Jersey; 6 IPÊ - Instituto de Pesquisas Ecológicas, Nazaré Paulista, São Paulo, Brazil; 7 Departamento de Biodiversidade, Laboratório de Primatologia, Instituto de Biociências, Universidade Estadual Paulista (UNESP), Rio Claro, São Paulo, Brazil; National Cheng Kung University, TAIWAN

## Abstract

Captive breeding programs have been used as a relevant strategy to maintain self-sustainable and demographically stable populations with the goal of safeguarding threatened species from their imminent risk of EXTINCTION. Thus, monitoring genetic diversity becomes essential to avoid the loss of genetic diversity and inbreeding depression throughout *ex situ* generations. Furthermore, such programs must carry out adequate metapopulation management to retain genetic diversity from the wild, minimizing eventual harmful effects associated with adaptation in captivity and sub-structuring. In this study, we analyzed *ex situ* populations of the endangered black lion tamarin (BLT), *Leontopithecus chrysopygus*, a primate endemic to the Brazilian Atlantic Forest. We monitored genetic diversity and structure in the three main *ex situ* groups for conservation purposes, before (2014) and after (2020) the transfer of five captive animals from Brazilian to European institutions. We also analyzed data from the whole studbook of the species to access life-history information about the *ex situ* populations. In addition, we performed an integrative *ex situ/in situ* analysis by including extant wild individuals from the same area of the founder population. Finally, we evaluated population viability based on genetic diversity trends predicted for the next 100 years. Our findings showed that the captive breeding program of BLT has been efficient in preventing the loss of heterozygosity despite significant reductions in allelic richness. This reduction is likely due to the loss of private and/or rare alleles resulting from the death of some individuals. The extant *ex situ* metapopulation and the wild population evidenced significant genetic differentiation and overall low levels of genetic diversity. The predictive analysis indicated that the loss of genetic diversity will be critical for the captive groups. However, the wild population demonstrated a greater capacity to retain genetic diversity over the next 100 years. These findings provide relevant information on the BLT’s captive breeding program and its founder-related wild population, as well as insights for further integrated *ex situ*/*in situ* management actions.

## Introduction

*Ex situ* conservation strategies have been extremely important in safeguarding rare and/or endangered species from their imminent risk of extinction [1–3]. In this context, captive breeding programs are primarily aimed at maintaining self-sustainable and demographically stable populations that retain the genetic diversity and evolutionary potential of the species [4–6], so they can eventually be used in reintroduction programs if necessary [7].

Despite the potential of captive breeding programs for conservation purposes, it is well known that *ex situ* populations face critical challenges due to the reduced number of founders and the consequent reduction in genetic diversity, as well as an increase in inbreeding during population growth [8]. On the other hand, in nature, threatened species also tend to present negative genetic consequences resulting from anthropogenic actions, such as habitat loss and fragmentation, hunting, and illegal trade [9,10]. Thus, bottleneck and strong genetic drift effects, reduction or even the absence of gene flow, and low levels of genetic diversity are commonly observed in small and fragmented wild populations [8,11]. This is the case of the black lion tamarin (BLT), *Leontopithecus chrysopygus* (Mikan, 1823), a Neotropical primate (Callitrichidae, Platyrrhini) endemic to the Brazilian Atlantic Forest in the state of São Paulo [12], a biome currently considered one of the most fragmented in the American continent [13].

The black lion tamarin is classified as endangered (EN) on the Red List of the International Union for Conservation of Nature and Natural Resources [14] and on the Brazilian official list of threatened species [15]. It was already considered extinct from 1905 to 1970 when a few wild individuals were found in the Morro do Diabo State Park, a protected area located in the Pontal do Paranapanema region, where the total population size was estimated at approximately 200 individuals at that time [12,16,17]. In 1984, the construction of a hydroelectric plant was initiated that would flood 10% of the Morro do Diabo State Park. This fact led to *in situ* conservation actions that included the intensification of field studies and the search for new individuals in the species distribution area [12,17]. Currently, the total population size in Morro do Diabo State Park is approximately 1200 BLTs. This is the largest wild population of the species, corresponding to 75–80% of the whole population size in nature, currently estimated at 1600–1800 individuals, which are distributed in 20 disconnected fragments of the Atlantic Forest in the São Paulo state [14,18,19].

At the time of rediscovery of the species, some individuals were brought to *ex situ* conditions to study their biology [12,17] and raise an insurance population to prevent the extinction of the species. The first captive group of BLTs was established in 1973 by the initial introduction of seven individuals (of which one died), from Morro do Diabo State Park to the Tijuca Biological Bank (Rio de Janeiro, RJ), which were transferred in 1979 to the newly created Primatology Center of Rio de Janeiro (CPRJ, Rio de Janeiro, Brazil) [12,20]. In the following decade, approximately 20 wild animals, rescued from the flooded area in the Morro do Diabo State Park, were integrated into the captive metapopulation, as follows: six individuals joined the CPRJ in November 1985, and 14 joined the Zoological Park Foundation of São Paulo (FPZSP, São Paulo, Brazil) in November 1986 [12,21].

To contribute to the management of the *ex situ* groups of BLTs, the International Recovery and Management Committee of the Black Lion Tamarin was created in 1987, an initiative that gave rise to the Studbook for the Black Lion Tamarin, which records all genealogical information for the whole *ex situ* population of the species [12]. Since then, the captive populations of BLTs have been managed mainly through studbook data analyses, which are carried out to inform recommendations for forming mate-pairs and supporting the species in *ex situ* conditions [22,23].

Studbook records are undoubtedly a relevant source of information that effectively contributes to captive breeding programs; however, studbooks may contain incomplete or missing data, especially for genetic relationships among founders [24,25]. Thus, more recently, *ex situ* management programs have been using molecular approaches to address issues related to unknown parentage, genetic diversity of founder individuals, and population structure, among others [23,26–28]. The inclusion of molecular data in captive breeding programs has provided relevant complementary information useful for *ex situ* management actions [23,28–31]. Furthermore, the One Plan Approach, an integrative strategy recommended by the IUCN Conservation Planning Specialist Group that includes both *ex situ* and *in situ* populations, should be carried out to allow a more comprehensive conservation plan and management of threatened species [32,33].

For the black lion tamarins, currently, three main institutions have been managing *ex situ* populations for conservation purposes: the FPZSP (São Paulo, Brazil), the CPRJ (Rio de Janeiro, Brazil), and the Durrell Wildlife Conservation Trust (DWCT, Jersey, Channel Islands). Recently, the viability of these populations was suggested to be critical since very few mate-pairs were able to reproduce in captivity conditions [22,23]. Thus, the captive breeding program recommended animal translocations among these institutions, accomplished in 2017 with the transfer of five individuals from CPRJ (N = 02) and FPZSP (N = 03) to DWCT, where there had been no reproduction since 2012 [22,23].

Overall, in the present study, we performed molecular analyses to assess the genetic diversity and population structure in *ex situ* populations of BLT before (2014) and after (2020) the mentioned management. We also analyzed data from the species’ studbook to access life-history information about the whole *ex situ* metapopulation and perform an integrative *ex situ*/*in situ* analysis by including wild animals from the Morro do Diabo State Park, i.e., the area of origin of the founder population. Finally, predictive analyses were implemented to simulate changes in genetic diversity over the next 100 years, considering bottleneck scenarios [34] and the potential of retaining 90% of the current genetic diversity in order to infer population viability [1]. We hypothesize that although the BLT captive breeding program is being conducted to avoid inbreeding and loss of genetic diversity, the viability of the *ex situ* metapopulation is compromised, given the small population size, the restricted number of mate-pairs and the scarcity of new wild animal introductions. In addition, due to the number of generations that have passed since the founder individuals went into *ex situ* condition, we do not expect that the extant *ex situ* populations still retain genetic diversity representative of the current wild population of Morro do Diabo State Park.

## Methods

### Ethical and legal requirements

Biological sample collections were performed following all recommendations proposed by the American Society of Primatologists (ASP) for the ethical treatment of non-human primates. Permits were issued by the Ethics Committee on Animal Experimentation of the Federal University of São Carlos (CEUA-UFSCar numbers 9805200815 and 7058110316); the Biodiversity Authorization and Information System of the Chico Mendes Institute for Biodiversity Conservation (SISBIO-ICMBIO numbers 36961, 34862 and 41375); the Registration and Research Management System of the Secretariat of Environment, Infrastructure and Logistics of São Paulo (CadGP process number 0014546/2022); and the National System of Genetic Heritage Management and Associated Traditional Knowledge (SISGEN number A411359 and A2FB428).

### Biological sampling and DNA extraction

In total, we molecularly analyzed 76 BLTs from wild (N = 11) and captive populations (N = 65). The captive populations included samples from the *ex situ* groups before (2014) and after (2020) management actions conducted by the captive breeding program of the species, according to the prior [35] and the latest [22] version of the BLT’s Studbook. Among the *ex situ* samples from 2014, 20 were from the Zoological Park Foundation of São Paulo (FPZSP), 17 were from the Primatology Center of Rio de Janeiro (CPRJ), and 16 were from the Durrell Wildlife Conservation Trust (DWCT), totaling 53 animals up to the 8^th^ generation (G8). Among the animals kept in *ex situ* conditions in 2020, we sampled 18 from FPZSP, 10 from CPRJ, and four from DWCT, totaling 32 animals up to the 9^th^ generation (G9). This sampling comprises an overlap of 20 individuals that were also sampled in 2014 (Table S1). Although the 2020 samples represent approximately 50% of the current captive population, they include the majority of active breeders and their offspring. This provides a proper representation of the genetic composition of the population. The wild samples (N = 11) came from Morro do Diabo State Park (−22.61212; −52.18244), a continuous patch of Atlantic Forest, where the wild founders (F0) originated [22]. Complementary information about the studied specimens, including sex and type of samples collected, is provided in S1 and S2 Tables.

For biological sampling, the individuals were manually captured and anesthetized following anesthetic protocols for chemical immobilization using a combination of ketamine (12 mg/kg) and midazolam (0.5 mg/kg), intramuscularly administered, or via direct induction using inhalation anesthesia equipment calibrated with Isoflurane (2–5%) and Oxygen (2L/min). Then, approximately 0.3 mL of fresh blood was collected using a vacutainer containing EDTA (3.6 mg). These procedures were performed by a veterinarian, who monitored and released the animals after sampling. When it was not possible to collect blood samples, hair tufts were pulled from the dorsal part of the animals and packed in plastic bags containing silica crystals. Blood and hair samples were stored at −20 °C and room temperature, respectively. Non-invasive fresh fecal samples from three non-captured wild animals were also collected and stored in absolute alcohol at −20^o^C.

### Studbook analyses

Pedigree records of the prior [35] and the latest [22] published versions of the BLT Studbook were analyzed to access the life-history of the animals kept in *ex situ* conditions and infer population sizes, sex ratio, and inbreeding coefficient - the latter estimated with PMx software version 1.8.0 [36], using data available in the studbook from 1973 to June 2020. We also depicted a genealogical diagram for the *ex situ* metapopulation of BLT from 2014 and 2020, considering the extant animals and their respective ascendent generations, using the Pedigraph software [37].

### Molecular analyses

DNA extractions were performed using the phenol protocol [38] for blood and hair samples and the Qiagen Stool Mini Kit (Qiagen, Hilden, Germany) for fecal samples. DNA integrity and quantity were evaluated using a Nanodrop spectrophotometer (NanoVue Plus, GE Healthcare, Chicago, USA). Polymerase Chain Reactions (PCRs) were performed for 12 microsatellite loci previously described for *L. chrysopygus* [39] and *Leontopithecus chrysomelas* [40] (Table 1).

**Table 1:** Summary information on the microsatellite loci analyzed, including the original species. F: forward and R: reverse. AT (ºC): alignment temperature. ABI: fluorochrome used.

Species	Locus	Primer Sequence (5’–3’)	AT (ºC)	ABI
*Leontopithecus chrysopygus*	Leon2	F: CTGCTTCTTGTTCCACTTCTTCTC R: GTTTGGGTGGTTGCCAAG	55	FAM
	Leon3c20	F: CTGTATGTGATCGCTTTTACCTG R: AAGGCAATCTAACTAATCAACACTC	60	NED
	Leon11c72	F: AGGATTACAGGTGCCCAC R: TTGCATATTGTGTTCAACTTC	60	VIC
	Leon15c85	F: CTGATCCTTGAAGCAGCATTG R: GGTTAAAGGGGTTCGTTCTGTG	60	FAM
	Leon21c75	F: CAGTTGAGGGAACAGGAATTA R: CACTGCACTGACAGAGCAAG	60	FAM
	Leon30c73	F: GGACCTGATTGAAGCAGTC R: TTCCCTGAGAATCTAATGGAG	60	NED
	Leon31c97	F: TGGTCCAGAGAAATGATGTC R: GTAATTCCTTGGATTTATGCC	58	PET
	Leon35c42	F: GTGGAAAGGTTTCAGAATATC R: TGCAGTTGTCCACACTTTA	60	FAM
*Leontopithecus chrysomelas*	Lchu01	F: GCTCAGGTGTTATTTATGTCCAAA R: GTTTCTTGCAACTATCTTGCATGTTCTGC	58	FAM
	Lchu06	F: GCCTTAATTAGCACCAGAACC R: GTTTCTTACCACTCCAAGCCTTCAGTA	55	PET
	Lchu07	F: TCTCATTTCTTCTCATGGACTC R: GTTTCTTCTTGACTCACAGCATGACCT	55	FAM
	Lchu08	F: CACGGCAATGTGGGAATAA R: GTTTCTTTTCAGTAGTTGGGACTGGGATAA	58	VIC

PCRs were carried out using a final reaction volume of 10 μL, containing 50 ng of DNA template, 0.40 pmol of each reverse and M13 primer, 0.10 pmol of the forward primer, 1x GoTaq (Promega, Madison, United States), containing 1x Taq buffer and 1.5 mM of MgCl_2_, 0.30 mg/ml of BSA, 0.25 mM of dNTPs, and an additional of 0.75 mM of MgCl_2_ for DNA from blood samples, and 1.50 mM for DNA from hair or fecal samples. PCRs were carried out on an Eppendorf Mastercycler Gradient® Thermal Cycler (Eppendorf AG, Hamburg, Germany) under the following conditions: 1º step: 5 minutes at 94 °C for initial denaturation; 2º step: 35 cycles of 30 seconds at 94 °C for denaturation, 45 seconds at the primer-specific annealing temperature indicated in Table 1, and extension for 45 seconds at 72 °C; 3º step: 10 cycles of 30 seconds at 94 °C, 45 seconds at 53 °C (annealing temperature of M13 primer), and 45 seconds at 72 °C; 4º step: 10 minutes at 72 °C for the final extension step.

Amplification patterns were first verified by electrophoresis using a 2% agarose gel under constant voltage (100 V for 35 min) on a transilluminator (ETX-35.M, Vilmer Lourmat, Collégien, France). Then, genotyping runs were performed on an ABI3730XL automatic sequencer (Applied Biosystems, Foster City, United States) using the GS 500 Liz size standard. Electropherograms were analyzed, and alleles and genotypes were scored using Geneious 6.0.6 [41]. Genotyping errors were checked by duplicates of homozygous alleles, and no genotyping errors regarding preferential amplification of alleles were observed, independently of the sample type. Because DNA from hair and fecal samples had lower amplification rates than those from blood samples, PCRs for hair and fecal samples were performed three and four times, respectively. The PCR products were genotyped at least twice and three times for hair and fecal samples, respectively, to avoid genotyping errors and ensure accuracy of allele scoring. We found a higher rate of genotyping failure for fecal (37.7%) and hair (33.3%) samples, compared to blood samples (14.7%).

### Genetic diversity and structure analyses

The occurrence of null alleles, allelic dropout, and stuttering for all scored alleles was verified using the software Micro-Checker [42]. Hardy-Weinberg Equilibrium (HWE) deviations were tested using Genepop 1.2 [43]. Polymorphic Information Content (PIC) values were determined with Cervus 3.0.3 [44], and the number of alleles (A_N_), and expected (H_E_) and observed (H_O_) heterozygosity were estimated using GenAlex 6.3 [45]. Allelic richness (A_R_) and inbreeding coefficient (F_IS_) were determined with FSTAT 2.9.3.2 [46]. To compare genetic diversity parameters (H_E_, H_O,_ F_IS,_ and A_R_) between the years, *P*-values for significant differences (*P* < 0.05) were calculated using the Mann-Whitney test following the assessment of normality and homogeneity of the sampling, as determined by the Shapiro-Wilk and Levene tests, respectively, in R 4.3.1 [47].

Population structure was also examined through Principal Coordinate Analysis (PCoA), using GenAlEx 6.4 [45], and the Bayesian clustering method implemented with Structure 2.1 [48]. The structure analysis was performed without prior information, considering ten replicates of each run, for K values ranging from 1 to 4, using the admixture model with a MCMC of 200,000 interactions and a burn-in period of 40,000 sets. We determined the number of genetic groups using the optimal posterior probability value, given the modal value of ΔK [49]. K statistics were generated using Structure Harvest [50]. In addition, we performed pairwise comparisons of G_ST_ [51] and Jost’s D [52] in the DEMEtics package [53].

Recent population reductions were assessed using the Bottleneck 1.2.02 program [54] through Wilcoxon’s test, which is commonly employed in studies using small sample sizes and less than 20 polymorphic loci [55]. The analyses were performed using the TPM model with 95% single-step mutations, 5% multiple-step mutations, and a variance among multiple steps of 12 [55].

Predictive analyses were performed with Bottlesim 2.6 [34], using the allele frequencies and the estimated population size (Ne), which was calculated using the linkage disequilibrium through the NeEstimator 2.0 software [56]. When it was not possible to calculate Ne values (S5 Table), the population size (N) was used. To infer population viability, we considered the chance of retaining 90% of the current genetic diversity [8] in the next 100 years under scenarios for population sizes of 100%, 75%, 50%, and 25%. The predictive analyses were performed with 10,000 iterations and considered a single bottleneck event to simulate population reductions and changes in heterozygosity and allele number. We assumed that *L. chrysopygus* reaches sexual maturity at 2 years of age and presents complete overlapping of generations, dioecious reproduction, a sex ratio of 1 female:1.5 male, and that its lifespan is 16 years [57–59].

## Results

### Pedigree data

The pedigree records for the whole *ex situ* metapopulation of BLT revealed a total of 559 animals spanning from F0 to G9, with F0 representing the founder individuals and G1-G9 representing generations raised in captivity. The first generation (G1) comprises offspring born in captivity from 22 wild animals (F0). The subsequent generations include individuals that were born mainly in captivity and eventually that came from the wild. Between 1973, when the *ex situ* population of BLT was established, and 1987, when the BLT Studbook was initiated, 27 animals from Morro do Diabo State Park were introduced in captivity. After this period, the introduction of wild animals became very limited, occurring only opportunistically, and involved the *ex situ* allocation of merely 11 rescued wild animals, as follows: three from Morro do Diabo State Park; three from Ribeirão Bonito Farm, a private fragment located nearby Morro do Diabo State Park; one from Buri, a municipality in the Upper Paranapanema region, in southeast São Paulo; and four with unknown origin.

**Fig 1:**
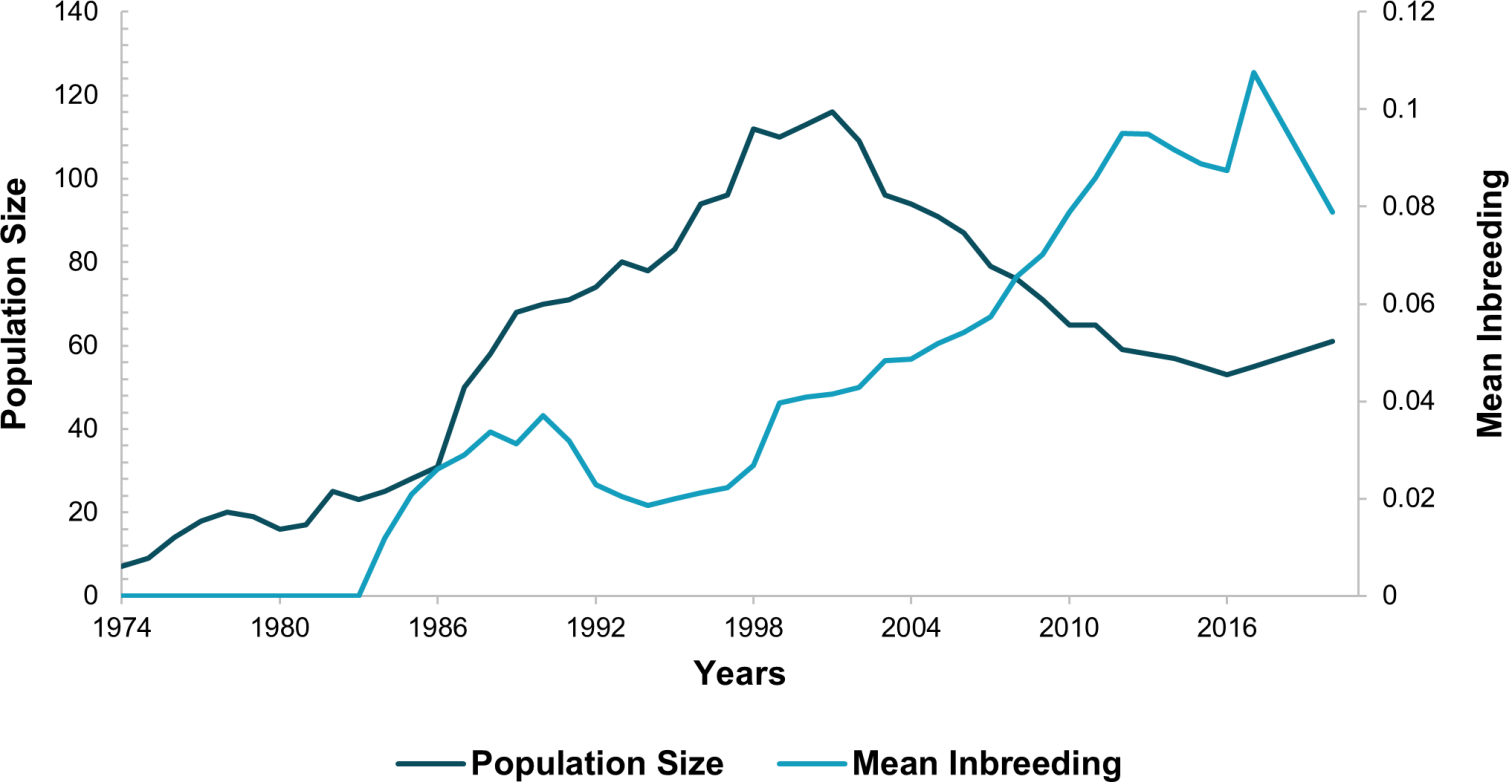
Population size and average inbreeding per year in captivity. The light blue line represents the estimated value of the pedigree inbreeding coefficient (F), and the dark blue line represents the population size.

The *ex situ* metapopulation of BLT in 2014 consisted of 56 animals spanning from G5 to G8, including two wild individuals (#430 from Buri, that entered the captive population in 2007, and #464 from Morro do Diabo State Park, which entered in 2010). Both wild individuals reproduced, contributing to the population’s genetic pool (S1 Fig). In total, eight individuals were actively reproducing at that time. In 2020, the *ex situ* metapopulation presented 61 animals from G6 to G9 generations (S2 Fig), with 10 actively reproducing individuals. From this total, only two breeders (#430 and #464) were from the wild. Despite the increase in population size over one generation, the overall population size of the *ex situ* metapopulation has exhibited a continuous decline from 1996 to 2016 (Fig 1).

According to the studbook records, the overseas population of DWCT experienced a significant reduction in size, decreased from 10 animals in 2014 to just four animals in 2017, when management actions occurred. After translocations of two males and three females from CPRJ and FPZSP to DWCT in 2020, the population size at DWCT was 10 (four males and five females, and one with unrecorded sex). Furthermore, a couple consisting of a female from CPRJ (#487) and a male from FPZSP (#472) successfully reproduced, marking the first offspring abroad after approximately five years without births. In FPZSP, the population increased from 21 (2014) to 33 (2020) individuals, comprising 17 males and 13 females. In contrast, the total population size in CPRJ declined from 17 (2014) to 10 (2020), with eight males and only two females. In addition to the translocation of the five animals from both Brazilian institutions (FPZSP and CPRJ) to DWCT, new *ex situ* groups were established in three other Brazilian institutions (Pomerode Zoo, Santa Catarina; Belo Horizonte Zoo, Minas Gerais; Onça Pintada Breeding, Paraná). However, only six couples reproduced regularly, during the period (three in FPZSP, one in DWCT, one in CPRJ, and one in the new *ex situ* population founded in the Pomerode Zoo), with no births recorded in CPRJ since 2016.

In addition to the small number of couples able to reproduce in captivity, there is still an unequal contribution to the gene pool of the offspring, with almost 50% of the extant animals in 2020 descending from just one couple kept in FPZSP, which has been actively breeding since 2010. Therefore, the captive metapopulation of BLT in 2020 is mostly descended from a wild female (#430) and a male (#412) born in captivity in 2003. This male descends from the founder population of Morro do Diabo State Park.

The pedigree-based inbreeding coefficient (F) has been increasing over the years, reaching its maximum value in 2017 (F = 0.107), when the population size was 55 individuals (Fig 1), before the translocation action. However, a comparison of inbreeding coefficients over the years revealed that the F value in 2014 (F = 0.091) was higher than that in 2020 (F = 0.078), suggesting that the recent management has been efficient in preventing an increase in inbreeding during the period (Fig 1).

### Genetic diversity and structure monitoring

The microsatellite panel used in this study was classified as moderately informative [60], according to the mean PIC value estimated for the captive (0.361) and wild samples (0.294) as well as for all samples combined (0.407). A total of 35 alleles were identified across captive and wild populations, with a mean number of 2.91 alleles per locus, ranging from two to four (S3 Table). No evidence of genotyping errors, null alleles, allele dropout, or stuttering was detected for any loci used in this study. However, HWE deviations were observed in some populations, as shown in S3 Table.

The genetic diversity estimates for the *ex situ* populations revealed expected heterozygosity ranging from 0.387–0.459 (2014) to 0.434–0.468 (2020) and observed heterozygosity ranging from 0.625–0.770 (2014) to 0.618–0.701 (2020). The number of alleles ranged from 24–26 (2014) to 25 (2020), while the mean allelic richness varied from 1.938–2.078 (2014) to 1.908–2.039 (2020), showing significant differences in FPZSP and CPRJ between both periods (Mann-Whitney test: p < 0.0001 and p < 0.001, respectively). However, the expected and observed heterozygosity values were not significantly different among the three institutions over the years (Table 2). The mean observed heterozygosity was higher than the expected heterozygosity, indicating negative inbreeding coefficients (F_IS_) in all captive populations analyzed (S4 Table).

**Table 2:** Comparison of mean genetic diversity parameters (GD) estimated for two periods (2014 and 2020) for the *ex situ* populations of *Leontopithecus chrysopygus* from Primatology Center of Rio de Janeiro (CPRJ), the Zoological Park Foundation of Sao Paulo (FPZSP), and Durrell Wildlife Conservation Trust (DWCT), considering 12 microsatellite loci, and the P-value for significant differences (P < 0.05)^*^.

GD	FPZSP			CPRJ			DWCT		
	2014	2020	p-value	2014	2020	p-value	2014	2020	p-value
**H**_**O**_	0.625	0.618	0.9525	0.770	0.696	0.2158	0.669	0.701	0.9376
**H**_**E**_	0.437	0.439	0.777	0.459	0.468	0.5706	0.387	0.434	0.716
**A**_**R**_	2.002	1.908	0.00008^*^	2.078	1.985	0.00078^*^	1.938	2.039	0.3614
**F**_**IS**_	-0.408	-0.385	0.7191	-0.660	-0.445	0.1643	-0.707	-0.507	0.256

H_O_: observed heterozygosity, H_E_: expected heterozygosity, A_R_: allelic richness; F_IS_: Inbreeding coefficient.

*Statistically significant values.

**Fig 2:**
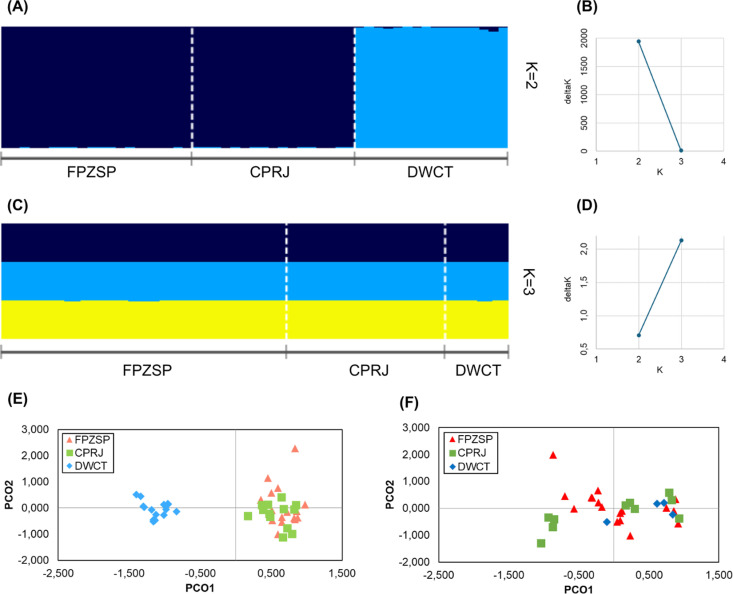
Population differentiation analyses of *ex situ* populations of *Leontopithecus chrysopygus* from the Zoological Park Foundation of São Paulo (FPZSP), Primatology Center of Rio de Janeiro (CPRJ), and Durrell Wildlife Conservation Trust (DWCT) in the years 2014 and 2020. A: Structure analysis in 2014; B: Values of Delta K for the Structure analysis in 2014; C: Structure analyses in 2020; D: Values of Delta K for the Structure analysis in 2020; E: Principal coordinate analysis (PCoA) showing the scores on the first (PCO1) and second (PCO2) principal coordinates in 2014; F: Principal coordinate analysis (PCoA) showing the scores on the first (PCO1) and second (PCO2) principal coordinates in 2020.

The Bayesian structure analysis indicated that DWCT was genetically differentiated from both CPRJ and FPZSP in 2014 (Figs 2A and 2B). However, after the translocations, all captive groups exhibited a similar probability of belonging to a single genetic cluster in 2020 (Fig 2C). Although Evanno’s criterion [49] suggested K = 3 as the most likely (Fig 2D), the individual assignment plot indicated a similar probability of an individual belonging to any of the three *ex situ* groups, supporting the absence of population structuring. The PCoA also evidenced two clusters in 2014 (Fig 2E) and a single cluster in 2020 (Fig 2F). Pairwise comparisons of Jost’s D and G_ST_ revealed significant differentiation between FPZSP and DWCT and between CPRJ and DWCT in 2014, but no significant differentiation was observed in 2020 (Table 3), corroborating the results of the previous analyses.

**Table 3:** Pairwise D-Jost (below the diagonal) and G_ST_ (above the diagonal) among the *ex situ* groups of *Leontopithecus chrysopygus* from Zoological Park Foundation of São Paulo (FPZSP), Primatology Center of Rio de Janeiro (CPRJ), and Durrell Wildlife Conservation Trust (DWCT) in 2014 and 2020.

2014	FPZSP	CPRJ	DWCT
FPZSP	–	0.007 (0.321)	0.130^*^ (0.003)
CPRJ	0.005 (0.746)	–	0.120^*^ (0.003)
DWCT	0.322^*^ (0.003)	0.322^*^ (0.003)	–
2020	**FPZSP**	**CPRJ**	**DWCT**
FPZSP	–	0.003 (0.561)	0.009 (NA)
CPRJ	0.002 (1.000)	–	-0.001 (0.999)
DWCT	0.019 (0.485)	-0.011 (1.000)	–

*Significant values. Values after the Bonferroni correction are in parentheses.

### *Ex situ* and *in situ* integrative analyses

**Fig 3:**
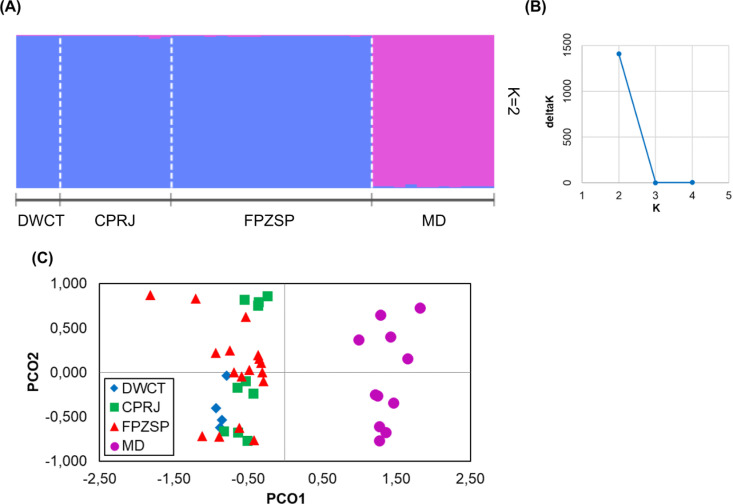
Integrative population structure analyses for *Leontopithecus chrysopygus* of the wild population from Morro do Diabo State Park (MD) and the captive populations from Zoological Park Foundation of São Paulo (FPZSP), Primatology Center of Rio de Janeiro (CPRJ), and Durrell Wildlife Conservation Trust (DWCT), according to Marques and Wormell (2020). A: Structure analysis (K = 2); B: Values of Delta K for the Structure analysis; and C: Principal coordinate analysis (PCoA), showing the scores on the first (PCO1) and second (PCO2) principal coordinates.

When we analyzed the extant *ex situ* populations (2020) and the wild population from Morro do Diabo State Park in an integrative manner, we identified two main genetic clusters (K = 2) through both Bayesian inference (Figs 3A and 3B) and Principal Coordinate Analysis (Fig 3C), clearly distinguishing the *ex situ* groups from the wild population. This result was confirmed by both D-Jost and G_ST_ (Table 4).

**Table 4:** Pairwise D-Jost and G_ST_ among the wild population of *Leontopithecus chrysopygus* from Morro do Diabo State Park (MD) and the *ex situ* populations in 2020, considering the single ones - FPZSP (Zoological Park Foundation of São Paulo), Primatology Center of Rio de Janeiro (CPRJ), Durrell Wildlife Conservation Trust (DWCT) - and the metapopulation.

Ex situ population in 2020	G_ST_	p-value	D-Jost	p-value
FPZSP	0.169	0.001 (0.003)^*^	0.320	0.001 (0.006)^*^
CPRJ	0.155	NA	0.297	0.001 (0.006)^*^
DWCT	0.172	NA	0.381	0.001 (0.006)^*^
Metapopulation	0.162	0.001 (0.001)^*^	0.320	0.001 (0.001)^*^

*Significant values. Values after the Bonferroni correction are in parentheses. NA: Not available.

**Fig 4:**
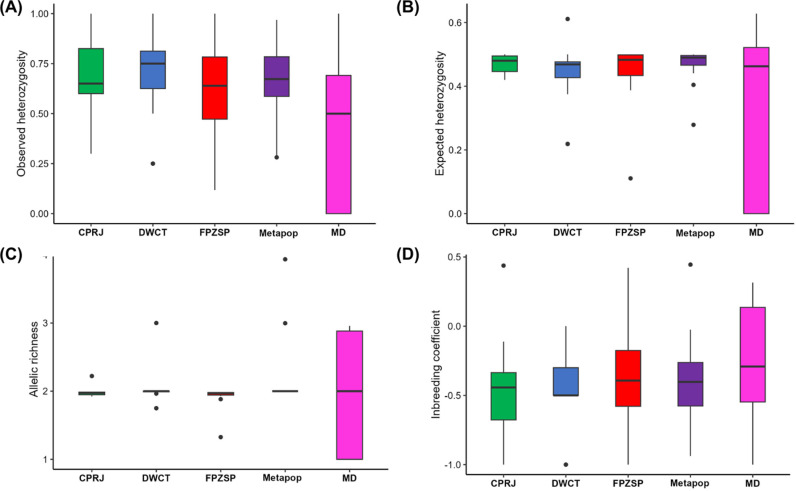
Integrative genetic diversity analyses for *Leontopithecus chrysopygus,* showing the boxplot for expected heterozygosity (H_E_), allelic richness (A_R_), observed heterozygosity (HO), and inbreeding coefficient (F_IS_) in wild populations from Morro do Diabo State Park (MD) and in captive populations from Zoological Park Foundation of São Paulo (FPZSP), Primatology Center of Rio de Janeiro (CPRJ), and Durrell Wildlife Conservation Trust (DWCT) and the whole captive metapopulation (Metapop), according to Marques and Wormell (2020).

The expected heterozygosity, observed heterozygosity, inbreeding coefficients and allelic richness were not significantly different between the extant (2020) whole captive metapopulation (H_E_ = 0.462; A_R_ = 2.244; H_O_ = 0.652; F_IS_ = -0.397) and the sampled wild population, which showed H_E_ = 0.347, H_O_ = 0.450, A_R_ = 2.043 and F_IS_ = -0.243 (Fig 4, S4 Table).

**Fig 5:**
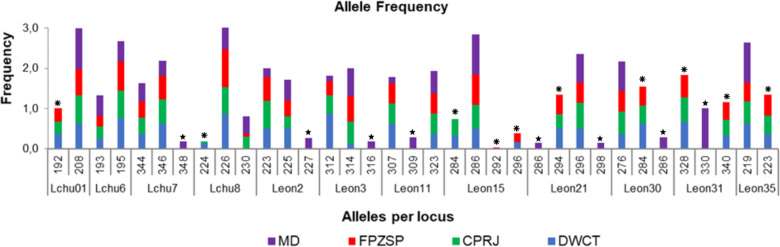
Allele frequency for the wild population of *Leontopithecus chrysopygus* from Morro do Diabo State Park (MD); and for the captive populations of Primatology Center of Rio de Janeiro (CPRJ), Zoological Park Foundation of São Paulo (FPZSP), and Durrell Wildlife Conservation Trust (DWCT), evidencing ten private alleles found in the *ex situ* populations (asterisk) and eight private alleles found in the wild population of Morro do Diabo State Park (star).

The total number of alleles (A_N_) observed in the captive and wild populations was 25, of which eight alleles were privates in the wild population and 10 alleles were privates in the captive population (Fig 5).

The Bottleneck analysis provided evidence of recent population declines in both the captive metapopulation and the wild population, consistently across all analyzed models (Table 5).

**Table 5:** P-values for the Bottleneck analysis in the captive metapopulation and the wild population from Morro do Diabo State Park of *Leontopithecus chrysopygus*, according to different mutation models: IAM (Infinite Allele Model), SMM (Stepwise Mutation Model), and TPM (Two-Phase Mutation Model).

Captive metapopulation	I.A.M.	T.P.M.	S.M.M.
Heterozygosity deficiency	0.99976	0.99915	0.99829
Heterozygosity excess	0.00037^*^	0.00122^*^	0.00232^*^
Heterozygosity excess and deficiency	0.00073^*^	0.00244^*^	0.00464^*^
**Morro do Diabo State Park**	**I.A.M.**	**T.P.M.**	**S.M.M.**
Heterozygosity deficiency	0.99805	0.98633	0.98633
Heterozygosity excess	0.00391^*^	0.01953^*^	0.01953^*^
Heterozygosity excess and deficiency	0.00781^*^	0.03906^*^	0.03906^*^

*Significant values: p < 0.05.

### Predictive analyses for genetic diversity trend

**Fig 6:**
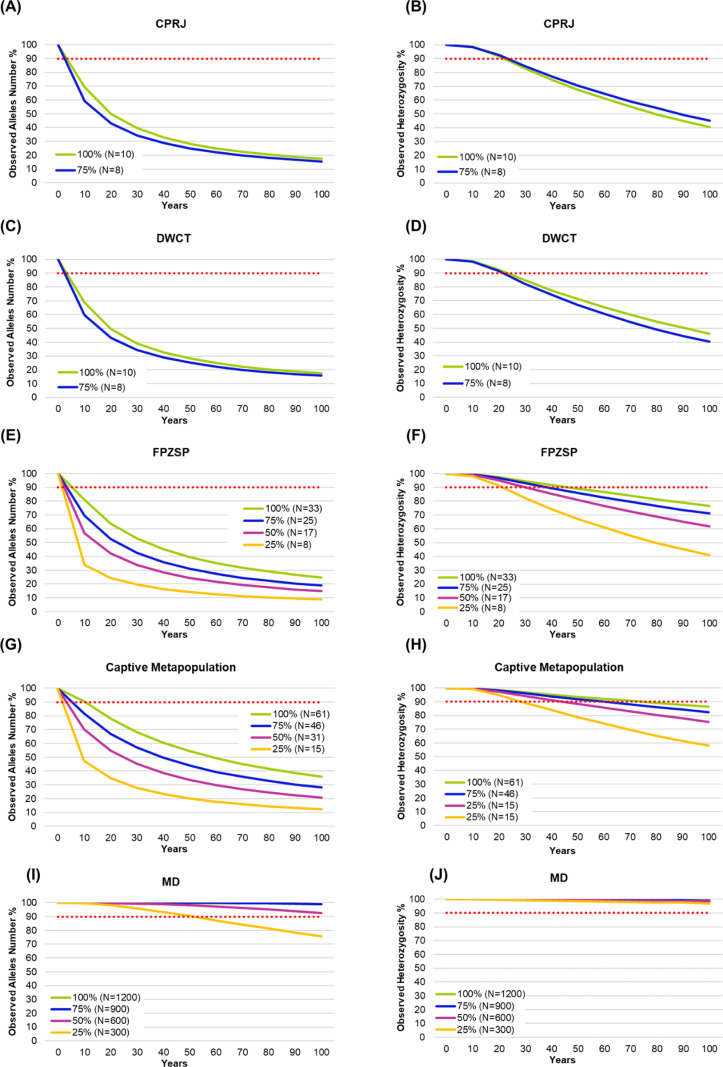
Prediction analysis for observed allele number and observed heterozygosity reductions of the *ex situ* populations of *Leontopithecus chrysopygus* (2020) from the Primatology Center of Rio de Janeiro (CPRJ) (A-B), Durrell Wildlife Conservation Trust (DWCT) (C-D) and the Zoological Park Foundation of São Paulo (FPZSP) (E-F); the captive metapopulation (G-H), and the wild population of Morro do Diabo State Park (MD) (I-J) for the next 100 years, using 100% of the population sizes and bottlenecks of 25%, 50%, and 75%.

When we simulated the trend of genetic diversity for the *ex situ* groups in 2020, as well as for the wild population, considering the total and the estimated population sizes, respectively, we observed that the predicted bottleneck scenarios evidence a tendency for reductions in genetic diversity likely explained by the reduction in population sizes (Fig 6). The prediction for genetic diversity reduction is quite dramatic in all captive populations, even when analyzed as a single larger metapopulation. Under bottleneck scenarios the captive metapopulation will be able to retain 90% of the current genetic variation for only 10–15 years. If no bottleneck occurs, the metapopulation will retain 90% of the current genetic diversity for approximately 20 years. For the wild population, it will be able to retain 90% of the current genetic diversity in most of the bottleneck scenarios tested, except for the number of alleles, which will decrease in the next 50 years if there is a 75% reduction in its population size.

**Fig 7:**
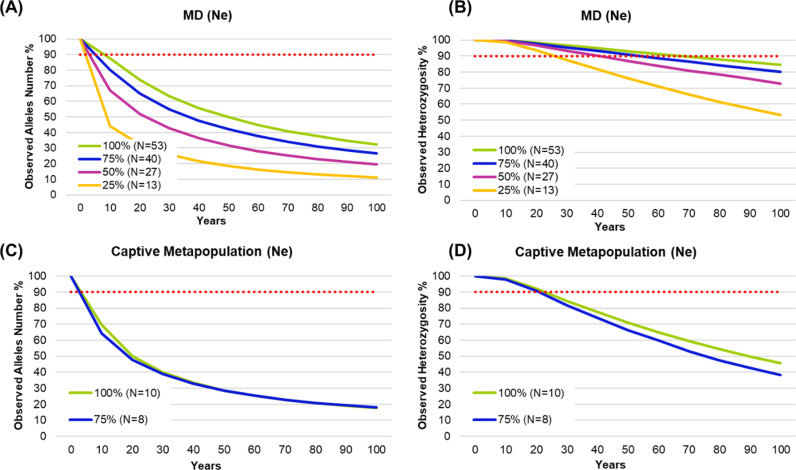
Prediction analysis for observed allele number and observed heterozygosity reductions of the wild population of Morro do Diabo State Park (MD) (A-B) and the whole *ex situ* metapopulation of *Leontopithecus chrysopygus* (2020) (C-D) for the next 100 years, using 100% of the effective population sizes (Ne) and bottlenecks of 25%, 50%, and 75%.

The scenario becomes much more critical if we consider the Ne values (S5 Table). Under bottlenecks, heterozygosity of the wild population will be retained for 30–70 years, while the number of alleles will drop to less than 90% in the next 10 years in all simulated scenarios (Figs 7A and 7B). For the captive metapopulation, the loss of genetic diversity will be more pronounced (Figs 7C and 7D).

## Discussion

Our study was able to assess the genetic diversity and structure in *ex situ* populations of black lion tamarins before (2014) and after (2020) recent management actions, which transferred five captive animals from Brazil to European captivity, as well as in wild extant individuals from Morro do Diabo State Park, i.e., area from where the captive founders originated. In addition, through simulation analyses, we evaluated scenarios to predict the retention of genetic diversity over the next 100 years and analyzed inbreeding based on pedigree data.

The pedigree analysis of data from 2014 to 2020 showed that the captive breeding program of BLT was able to prevent increased inbreeding during the period. The average inbreeding rate (F) in 2020 was lower compared to 2014. Evidence of inbreeding depression, such as bone deformities, low sperm motility, high infertility rates, cleft lips, and a high incidence of gallbladder illness, had been observed in the European captive population of BLT, before the recent management actions [23]. In this sense, the transfer of individuals from FPZSP and CPRJ to DWCT allowed the formation of new mate-pairs and viable litters abroad [22], overcoming the previous inbreeding depression effects. Furthermore, molecular analyses indicated changes in population structure but very little variation in genetic diversity parameters between 2014 and 2020.

We did not find significant differences among the heterozygosity values from both periods, only in the allelic richness, that showed a low decrease in both CPRJ and FPZSP. This reduction is likely due to the loss of private and/or rare alleles resulting from the death of some individuals, in addition to the reproduction of a limited number of mate-pairs. Significant reductions in allelic richness, but none in observed heterozygosity, are expected, as allelic richness, compared to heterozygosity, is more prone to diversity loss in small populations, especially those originating from a narrow genetic base resulting from the founder effect [29,61].

Despite the presence of few private alleles in all captive groups, the genetic structure for the *ex situ* metapopulation shows no evidence of substructuring in 2020, in contrast to the results in 2014, which showed substructuring. Although genetically differentiated populations contribute to the total genetic diversity of a species, maintaining unique and sub-structured populations with a small number of individuals, as in the case of DWCT, increases the risk of genetic diversity loss, as such populations are more susceptible to stochastic events, inbreeding depression and/or local extinction [11,62,63].

The management actions, which transferred five captive animals from Brazil to European captivity, can explain these findings, reinforcing the success of such action. On the other hand, several private alleles were observed between the wild and captive populations, indicating a marked genetic differentiation. This result can be attributed primarily to genetic drift, given that the captive population was initially established with a small number of wild individuals from the same area (i.e., Morro do Diabo). Furthermore, adaptation to captivity is recognized as an important evolutionary force [11,62], and although we assessed genetic diversity using 12 neutral loci, it may influence the genome as a whole [64] and the presented scenario, especially considering that the *ex situ* metapopulation of this species went through nine generations with limited mate-pairs and input of genetic diversity from the wild.

The wild population studied here also evidenced a low level of genetic diversity. Several studies on small and isolated wild populations of endangered species, including the lion tamarins [27,65–68], have shown a common tendency of low levels of genetic diversity. In nature, strong bottlenecks and the absence of connectivity among fragments may result in genetic diversity being maintained at levels as low as those evidenced here in captive populations (H_E_ = 0.442–0.459). Indeed, the current genetic diversity for the wild population of Morro do Diabo State Park (H_E_ = 0.347) analyzed herein is low, and despite the small sample size analyzed, this value is comparable to those from other BLT wild populations (H_E_ = 0.295–0.403) previously studied using a similar set of microsatellites [27,39], supporting these findings.

Under captive conditions, the low genetic diversity can be explained by the founder effect and the reduced number of mate-pairs raising offspring, in addition to their unequal contributions [23]. In accordance with this statement, negative F_IS_ values can indicate the occurrence of bottlenecks in both wild and *ex situ* populations. The excess of observed heterozygotes and the low levels of genetic diversity, as obtained here, have already been associated with recent population reductions [1,69], a result that was also confirmed in this study after performing the bottleneck analysis. In addition, in both *ex situ* and *in situ* conditions, the absence of the input of new gene pools, by the lack of effective dispersal in the wild or lack of introduction of new unrelated animals in captivity, contributes to decreased genetic diversity and increased inbreeding [8,11].

Trying to understand population viability of both wild and *ex situ* populations, our simulation analyses evidenced that the wild population of Morro do Diabo State Park shows a good potential to retain genetic diversity throughout the next 100 years if no other bottleneck occurs. Nevertheless, if a significant bottleneck reduces the population size by 75%, the number of alleles will decline more rapidly, putting the viability of this population at risk within the next 50–60 years. In contrast, all *ex situ* populations will experience a drastic reduction in allele number within the next 5–10 years, even without bottlenecks. For heterozygosity, CPRJ and DWCT are at risk of collapse if their population sizes are reduced by 25–50%. However, if population sizes are maintained at 75–100%, the scenario becomes less critical, with CPRJ, DWCT, and FPZSP retaining proper heterozygosity over the next 20–30 and 50 years, respectively.

Retention of genetic diversity in the long term is crucial to ensure population viability, as it is linked to the potential to respond to environmental and climate changes, and, consequently, prevent the extinction risk [1,70,71]. This is especially critical in *ex situ* conditions, where selection pressure may be much stronger [62,70,72]. Notwithstanding the use of neutral markers, it is assumed that these markers are linked to non-neutral regions of the genome and, therefore, to the adaptive potential of populations and species [11,73–75]. In this sense, allelic diversity has been considered a relevant parameter in management and conservation programs [76,77], as it is deemed a valuable indicator of the evolutionary potential of populations and species [70]. Thus, even small, significant reductions in allelic diversity raises concern, as it may promote reductions in the potential for adaptation to eventual environmental changes [78,79].

One of the most effective strategies for retaining diversity in captive breeding programs is to minimize kinship between individuals, using pedigree data and mean kinship values to choose the ideal breeders [4,80–82]. More recently, molecular data have also helped decision-making for the proper management of *ex situ* populations of lion tamarins, by suggesting the choice of animals with smaller mean kinship and greater individual heterozygosity [27] and indicating populations with lower levels of genetic diversity and higher risk to collapse [27,28]. In this sense, our findings emphasize the importance of an integrative metapopulation management action, including all institutions that maintain the species under human care for conservation purposes and, if possible, wild populations as well, as proposed by the One Plan Approach [32,33].

Overall, based on molecular genetic parameters and predictive analyses, introducing new gene pools from the wild into captivity is necessary to improve the management and genetic health of the *ex situ* metapopulation. This approach may help establish an insurance population [83] that can be used for reintroductions into the wild if necessary, and thus to prevent species’ extinction. The introduction of less related and more diverse animals will certainly bring great benefits if they successfully reproduce under *ex situ* conditions and contribute to form new mate-pairs with captive animals. However, this task is challenging, since the wild population is very small, and the removal of even a few animals or even a single couple could significantly impact the social group and local population. On the other hand, fires, deforestation and illegal trafficking continue to threaten the species’ persistence in the wild. Therefore, to indeed have an *ex situ* safeguard population, we recommend introducing new founders from the wild into captivity.

Alternatively, in addition to pedigree-based scores, the diversity of alleles and genotypes currently available in *ex situ* condition may be considered in management decisions. To this end, we suggest an effort to include a greater number of polymorphic loci and of analyzed animals in further molecular approaches. Exchanges between the three institutions should be prioritized, particularly between FPZSP and CPRJ, given that CPRJ has only two non-reproductive females; and then from both to DWCT, considering the still small population size in Europe. In this sense, recommendations for forming new potential mate-pairs should take into account, beyond the pedigree data, individual heterozygosity and allele differences among captive individuals, aiming to promote the reproduction of less homozygous animals, with greater allele diversity.

Although it has been a difficult task to survey forest patches and track/monitor these small endangered monkeys, it is worth mentioning that much field effort has been devoted to collecting a larger sample size in Morro do Diabo State Park and other areas where the species occurs in the wild. Further genetic studies, including sampling of remaining wild populations throughout the species’ current range, as well as a broader representative sampling of the population in Morro do Diabo State Park, are necessary to better understand genetic diversity in the wild and to guide the introduction of individuals in captivity. Such efforts will provide valuable information for integrated *ex situ*/*in situ* management actions. These recommendations highlight the importance of integrated management for BLT, reinforcing the relevance of actions involving both captive and wild populations, and the use of molecular and pedigree data in order to safeguard the genetic health and long-term viability of the species.

## Supporting information

S1 Fig Representation of the genealogy of *Leontopithecus chrysopygus* in 2014, showing the extant individuals colored in yellow and their respective ascendent generations based on data from the species’ pedigree records. **Circles**, squares, and diamonds indicate, respectively, females, males, and unknown sex individuals. The lines connect the offspring to their respective sire and dam; and † indicates dead individuals. F0 represents the founder individuals, and G1 to G9 represents the subsequent captive generations.
(DOCX)

S2 Fig Representation of the genealogy of *Leontopithecus chrysopygus* in 2020, showing the extant individuals colored in yellow and their respective ascendent generations based on data from the species’ pedigree records. Circles, squares, and diamonds indicate, respectively, females, males, and unknown sex individuals. The lines connect the offspring to their respective sire and dam; and † indicates dead individuals. F0 represents the founder individuals, and G1 to G9 represents the subsequent captive generations.
(DOCX)

S1 Table Information related to the samples from Primatology Center of Rio de Janeiro (CPRJ); Zoological Park Foundation of São Paulo (FPZSP) and Durrell Wildlife Conservation Trust (DWCT) in 2014 analysis and 2020 analysis: Studbook number; location in 2014 and/or 2020 and sex. F indicate female; M indicate male, * indicate dead individuals or not sampled, ** indicate transferred individuals from the Brazilian to European captivity.
(DOCX)

S2 Table Information related to the wild population samples from Morro do Diabo State Park: Identification number (sample), sex, collection date, age, and type of sample collected. (DOCX)

S3 Table Genetic diversity estimates for expected heterozygosity (H_E_), allelic richness (A_R_), observed heterozygosity (H_O_), and inbreeding coefficient (F_IS_) for captive populations of *Leontopithecus chrysopygus* from Primatology Center of Rio de Janeiro (CPRJ), Zoological Park Foundation of Sao Paulo (FPZSP), Durrell Wild Conservation Trust (DWTC) in 2020, and wild population of Morro do Diabo State Park. (DOCX)

S4 Table Summary of the P-values for differences between the genetic diversity parameters of the captive and wild populations of *Leontopithecus chrysopygus.* CPRJ: Primatology Center of Rio de Janeiro; FPZSP: Zoological Park Foundation of São Paulo. Metapopulation: captive metapopulation. MD: Morro do Diabo State Park A_R_: alleles richness, H_E_: expected heterozygosity, H_O_: observed heterozygosity, F_IS_: inbreeding coefficient. * Significant statistical differences P < 0.05. p-adj: Values after the Bonferroni correction.
(DOCX)

S5 Table Summary of the effective population size (Ne) confidence interval (Parametric and JackKnife), estimated with NeEstimator 2.0 software, for the Brazilian captive and wild populations of *Leontopithecus chrysopygus.* (DOCX)
