# Supplementary material for: Genetic monitoring in ex situ populations of the endangered primate Leontopithecus chrysopygus and integrative analyses with the wild founder population
Source: PLoS One. 2025 May 7;20(5):e0322817. doi: 10.1371/journal.pone.0322817 (PMC12057915; doi:10.1371/journal.pone.0322817)
Supplement: S2 Fig — Circles, squares, and diamonds indicate, respectively, females, males, and unknown sex individuals. The lines connect the offspring to their respective sire and dam; and † indicates dead individuals. F0 represents the founder individuals, and G1 to G9 represents the subsequent captive generations. (DOCX) [file pone.0322817.s002.docx]

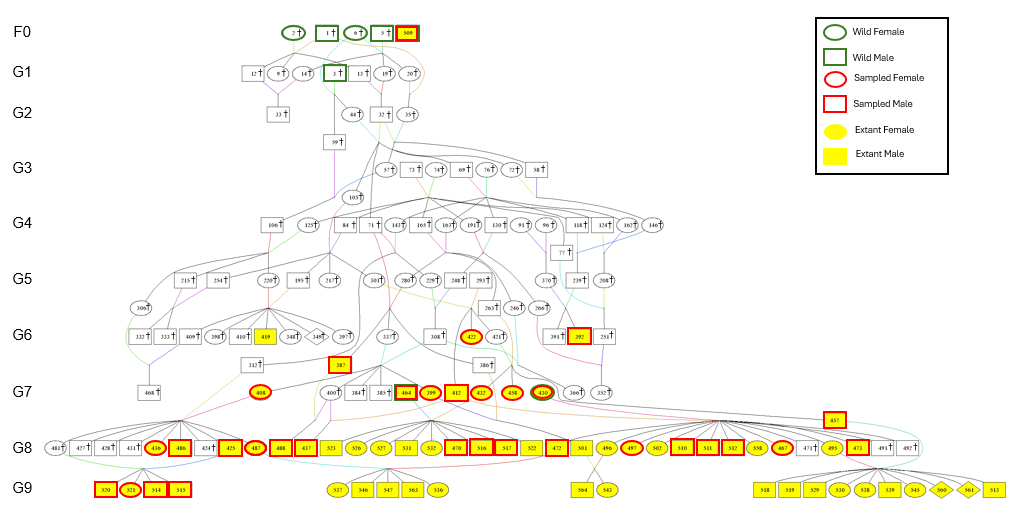


**S2 Fig. Representation of the genealogy of *Leontopithecus chrysopygus* in 2020, showing the extant individuals colored in yellow and their respective ascendent generations based on data from the species' pedigree records.** Circles, squares, and diamonds indicate, respectively, females, males, and unknown sex individuals. The lines connect the offspring to their respective sire and dam; and † indicates dead individuals. F0 represents the founder individuals, and G1 to G9 represents the subsequent captive generations.
