# Supplementary material for: Genetic monitoring in ex situ populations of the endangered primate Leontopithecus chrysopygus and integrative analyses with the wild founder population
Source: PLoS One. 2025 May 7;20(5):e0322817. doi: 10.1371/journal.pone.0322817 (PMC12057915; doi:10.1371/journal.pone.0322817)
Supplement: S1 Table — F indicate female; M indicate male, * indicate dead individuals or not sampled, ** indicate transferred individuals from the Brazilian to European captivity. (DOCX) [file pone.0322817.s003.docx]

**S1 Table. Information related to the samples from Primatology Center of Rio de Janeiro (CPRJ); Zoological Park Foundation of São Paulo (FPZSP) and** **Durrell Wildlife Conservation Trust (DWCT) in 2014 analysis and 2020 analysis: Studbook number; location in 2014 and/or 2020 and sex.**

| **Studbook number** | **2014 Analysis** | **2020 Analysis** | **2014 Location** | **2020 Location** | **Sex** |
| --- | --- | --- | --- | --- | --- |
| 110 | X | - (*) | DWCT |  | F |
| 125 | X | - (*) | DWCT |  | F |
| 189 | X | - (*) | DWCT |  | M |
| 195 | X | - (*) | DWCT |  | M |
| 220 | X | - (*) | DWCT |  | F |
| 256 | X | - (*) | DWCT |  | M |
| 261 | X | - (*) | DWCT |  | F |
| 263 | X | - (*) | FPZSP |  | M |
| 295 | X | - (*) | DWCT |  | M |
| 302 | X | - (*) | DWCT |  | F |
| 306 | X | - (*) | DWCT |  | F |
| 312 | X | - (*) | CPRJ |  | M |
| 320 | X | - (*) | DWCT |  | M |
| 327 | X | - (*) | DWCT |  | F |
| 366 | X | - (*) | CPRJ |  | F |
| 371 | X | - (*) | DWCT |  | M |
| 384 | X | - (*) | CPRJ |  | M |
| 385 | X | - (*) | FPZSP |  | M |
| 386 | X | - (*) | FPZSP |  | M |
| 387 | X | X | CPRJ | CPRJ | M |
| 391 | X | - (*) | FPZSP |  | M |
| 392 | X | X | FPZSP | FPZSP | M |
| 399 | X | X | FPZSP | FPZSP | F |
| 400 | X | - (*) | CPRJ |  | F |
| 408 | X | X | CPRJ | CPRJ | F |
| 409 | X | - (*) | DWCT |  | M |
| 412 | X | X | FPZSP | FPZSP | M |
| 420 | X | - (*) | DWCT |  | M |
| 421 | X | - (*) | FPZSP |  | F |
| 422 |  | X |  | FPZSP | F |
| 424 | X | - (*) | CPRJ |  | M |
| 425 | X | X | CPRJ | CPRJ | M |
| 427 | X | - (*) | CPRJ |  | M |
| 428 | X | - (*) | CPRJ |  | M |
| 430 | X | X | FPZSP | FPZSP | F |
| 431 | X | - (*) | CPRJ |  | M |
| 432 | X | X | FPZSP | FPZSP | M |
| 436 | X | X | CPRJ | DWCT (**) | F |
| 437 | X | X | CPRJ | CPRJ | F |
| 457 | X | X | FPZSP | FPZSP | M |
| 458 | X | X | FPZSP | FPZSP | F |
| 464 | X | X | FPZSP | FPZSP | M |
| 467 | X | X | FPZSP | FPZSP | F |
| 468 | X | - (*) | DWCT |  | M |
| 469 | X | - (*) | FPZSP |  | M |
| 470 | X | X | FPZSP | FPZSP | M |
| 471 | X | - (*) | FPZSP | DWCT (**) | M |
| 472 | X | X | FPZSP | DWCT (**) | M |
| 473 | X | X | FPZSP | FPZSP | M |
| 481 | X | - (*) | CPRJ |  | F |
| 486 | X | X | CPRJ | CPRJ | M |
| 487 | X | X | CPRJ | DWCT (**) | F |
| 488 | X | X | CPRJ | CPRJ | M |
| 497 |  | X |  | DWCT (**) | F |
| 500 | X | - (*) | FPZSP |  | U |
| 509 |  | X |  | FPZSP | M |
| 510 |  | X |  | FPZSP | M |
| 511 |  | X |  | FPZSP | M |
| 512 |  | X |  | FPZSP | M |
| 514 |  | X |  | CPRJ | M |
| 515 |  | X |  | CPRJ | M |
| 516 |  | X |  | FPZSP | M |
| 517 |  | X |  | FPZSP | M |
| 520 |  | X |  | CPRJ | M |
| 521 |  | X |  | CPRJ | F |

F – Female

M – Male

U - Unknow

(*) dead individuals or not sampled

(**) transferred individuals from the Brazilian to European captivity
