# Supplementary material for: Genetic monitoring in ex situ populations of the endangered primate Leontopithecus chrysopygus and integrative analyses with the wild founder population
Source: PLoS One. 2025 May 7;20(5):e0322817. doi: 10.1371/journal.pone.0322817 (PMC12057915; doi:10.1371/journal.pone.0322817)
Supplement: S2 Table — (DOCX) [file pone.0322817.s004.docx]

**S2 Table. Information related to the samples of wild population from Morro do Diabo State Park: Identification number (sample), sex, collection date, age and type of sample collected.**

| **Lab ID** | **ID** | **Collection date** | **Sex** | **Age** | **Sample** |
| --- | --- | --- | --- | --- | --- |
| MAM_0344 | MD1_01 | 10/11/2018 | Male | Adult | Hair |
| MAM_0345 | MD1_02 | 10/11/2018 | Male | Adult | Hair |
| MAM_0346 | MD1_03 | 10/11/2018 | Male | Adult | Hair |
| MAM_0347 | MD1_04 | 10/11/2018 | Female | Adult | Blood |
| MAM_1627 | MD1_A | 10/11/2018 | Male | Adult | Fezes |
| MAM_1628 | MD1_B | 10/11/2018 | NA | NA | Fezes |
| MAM_0433 | MD1_05 | 05/11/2019 | Female | NA | Blood |
| MAM_0434 | MD1_06 | 05/11/2019 | Male | Adult | Blood |
| MAM_0436 | MD1_08 | 05/11/2019 | Male | Adult | Blood |
| MAM_0438 | MD1_10 | 24/08/2019 | Male | Juvenile | Hair |
| MAM_0439 | MD1_11 | 24/08/2019 | Female | Adult | Hair |

NA: no available data.
