# Supplementary material for: Genetic monitoring in ex situ populations of the endangered primate Leontopithecus chrysopygus and integrative analyses with the wild founder population
Source: PLoS One. 2025 May 7;20(5):e0322817. doi: 10.1371/journal.pone.0322817 (PMC12057915; doi:10.1371/journal.pone.0322817)
Supplement: S3 Table — (DOCX) [file pone.0322817.s005.docx]

**S3 Table.** **Genetic diversity estimates for expected heterozygosity (H_E_), allelic richness (A_R_), observed heterozygosity (H_O_), and inbreeding coefficient (F_IS_) for captive populations of *Leontopithecus chrysopygus* from Primatology Center of Rio de Janeiro (CPRJ), Zoological Park Foundation of Sao Paulo (FPZSP), Durrell Wild Conservation Trust (DWTC) in 2020, and wild population of Morro do Diabo State Park.**

| **Locus** | **N** | **A_N_** | **A_E_** | **H_O_** | **H_E_** | **A_R_** | **F_IS_** | **P_DH_** | **P_EH_** | **P_HWE_** |
| --- | --- | --- | --- | --- | --- | --- | --- | --- | --- | --- |
| **Zoological Park Foundation of São Paulo** | | | | | | | | | | |
| **Lchu01** | 18 | 2.000 | 1.800 | 0.556 | 0.444 | 1.930 | -0.223 | 0.932 | 0.331 | 0.599 |
| **Lchu6** | 18 | 2.000 | 1.670 | 0.556 | 0.401 | 1.882 | -0.360 | 1.000 | 0.171 | 0.254 |
| **Lchu7** | 15 | 2.000 | 1.923 | 0.800 | 0.480 | 1.967 | -0.647 | 1.000 | 0.024* | 0.026* |
| **Lchu8** | 17 | 2.000 | 1.125 | 0.118 | 0.111 | 1.326 | -0.032 | 1.000 | 0.964 | 1.000 |
| **Leon2** | 18 | 2.000 | 1.946 | 0.722 | 0.486 | 1.970 | -0.464 | 0.996 | 0.071 | 0.075 |
| **Leon3** | 18 | 2.000 | 1.857 | 0.278 | 0.461 | 1.947 | 0.422 | 0.093 | 0.992 | 0.122 |
| **Leon11** | 18 | 2.000 | 2.000 | 1.000 | 0.500 | 1.981 | -1.000 | 1.000 | 0.001* | 0.000* |
| **Leon15** | 18 | 3.000 | 1.632 | 0.500 | 0.387 | 1.973 | -0.264 | 1.000 | 0.257 | 0.632 |
| **Leon21** | 18 | 2.000 | 2.000 | 1.000 | 0.500 | 1.981 | -1.000 | 1.000 | 0.001* | 0.000* |
| **Leon30** | 18 | 2.000 | 1.994 | 0.722 | 0.498 | 1.980 | -0.426 | 0.983 | 0.083 | 0.151 |
| **Leon31** | 18 | 2.000 | 1.976 | 0.778 | 0.494 | 1.976 | -0.556 | 0.997 | 0.025* | 0.051 |
| **Leon35** | 18 | 2.000 | 1.994 | 0.389 | 0.498 | 1.980 | 0.247 | 0.247 | 0.943 | 0.369 |
| **Mean** | **18** | **2.083** | **1.826** | **0.618** | **0.439** | **1.908** | **-0.385** | **1.000** | **0.001*** | **-** |
| **Primatology Center of Rio de Janeiro** | | | | | | | | | | |
| **Lchu01** | 10 | 2.000 | 1.724 | 0.600 | 0.420 | 1.922 | -0.385 | 1.000 | 0.339 | 0.482 |
| **Lchu6** | 10 | 2.000 | 1.724 | 0.600 | 0.420 | 1.922 | -0.385 | 1.000 | 0.333 | 0.482 |
| **Lchu7** | 10 | 2.000 | 1.923 | 0.800 | 0.480 | 1.975 | -0.636 | 1.000 | 0.094 | 0.171 |
| **Lchu8** | 10 | 3.000 | 1.942 | 0.600 | 0.485 | 2.222 | -0.187 | 0.804 | 0.376 | 0.433 |
| **Leon2** | 10 | 2.000 | 1.724 | 0.600 | 0.420 | 1.922 | -0.385 | 1.000 | 0.331 | 0.482 |
| **Leon3** | 10 | 2.000 | 1.980 | 0.300 | 0.495 | 1.986 | 0.438 | 0.207 | 0.979 | 0.244 |
| **Leon11** | 10 | 2.000 | 2.000 | 1.000 | 0.500 | 1.989 | -1.000 | 1.000 | 0.007* | 0.006* |
| **Leon15** | 10 | 2.000 | 1.923 | 0.800 | 0.480 | 1.975 | -0.636 | 1.000 | 0.072 | 0.173 |
| **Leon21** | 10 | 2.000 | 1.835 | 0.700 | 0.455 | 1.956 | -0.500 | 1.000 | 0.203 | 0.219 |
| **Leon30** | 10 | 2.000 | 1.980 | 0.900 | 0.495 | 1.986 | -0.800 | 1.000 | 0.032* | 0.046* |
| **Leon31** | 9 | 2.000 | 1.906 | 0.556 | 0.475 | 1.975 | -0.111 | 0.867 | 0.658 | 1.000 |
| **Leon35** | 10 | 2.000 | 1.980 | 0.900 | 0.495 | 1.986 | -0.800 | 1.000 | 0.024* | 0.046* |
| **Mean** | **10** | **2.083** | **1.887** | **0.696** | **0.468** | **1.985** | **-0.445** | **1.000** | **0.001*** | **-** |
| **Durrell Conservation Wild Trust** | | | | | | | | | | |
| **Lchu01** | 4 | 2.000 | 1.882 | 0.750 | 0.469 | 2.000 | -0.500 | 1.000 | 0.581 | 1.000 |
| **Lchu6** | 4 | 2.000 | 1.600 | 0.500 | 0.375 | 1.964 | -0.200 | 1.000 | 0.868 | 1.000 |
| **Lchu7** | 4 | 2.000 | 1.882 | 0.750 | 0.469 | 2.000 | -0.500 | 1.000 | 0.585 | 1.000 |
| **Lchu8** | 4 | 2.000 | 1.280 | 0.250 | 0.219 | 1.750 | 0.000 | 1.000 | 1.000 | - |
| **Leon2** | 3 | 2.000 | 2.000 | 1.000 | 0.500 | 2.000 | -1.000 | 1.000 | 0.421 | 0.396 |
| **Leon3** | 4 | 2.000 | 1.280 | 0.250 | 0.219 | 1.750 | 0.000 | 1.000 | 1.000 | - |
| **Leon11** | 4 | 2.000 | 1.882 | 0.750 | 0.469 | 2.000 | -0.500 | 1.000 | 0.572 | 1.000 |
| **Leon15** | 3 | 3.000 | 2.571 | 1.000 | 0.611 | 3.000 | -0.500 | 1.000 | 0.386 | 1.000 |
| **Leon21** | 4 | 2.000 | 2.000 | 1.000 | 0.500 | 2.000 | -1.000 | 1.000 | 0.232 | 0.317 |
| **Leon30** | 4 | 2.000 | 1.882 | 0.750 | 0.469 | 2.000 | -0.500 | 1.000 | 0.590 | 1.000 |
| **Leon31** | 3 | 2.000 | 1.800 | 0.667 | 0.444 | 2.000 | -0.333 | 1.000 | 0.818 | 1.000 |
| **Leon35** | 4 | 2.000 | 1.882 | 0.750 | 0.469 | 2.000 | -0.500 | 1.000 | 0.563 | 1.000 |
| **Mean** | **4** | **2.083** | **1.829** | **0.701** | **0.434** | **2.039** | **-0.507** | **1.000** | **0.010*** | **-** |
| **Morro do Diabo State Park** | | | | | | | | | | |
| **Lchu01** | 11 | 1.000 | 1.000 | 0.000 | 0.000 | 1.000 | - | - | - | - |
| **Lchu6** | 5 | 2.000 | 2.000 | 1.000 | 0.500 | 2.000 | -1.000 | 1.000 | 0.125 | 0.129 |
| **Lchu7** | 11 | 3.000 | 2.689 | 0.545 | 0.628 | 2.931 | 0.178 | 0.317 | 0.867 | 0.225 |
| **Lchu8** | 9 | 2.000 | 1.976 | 0.889 | 0.494 | 2.000 | -0.778 | 1.000 | 0.046* | 0.058 |
| **Leon2** | 11 | 3.000 | 2.659 | 0.455 | 0.624 | 2.958 | 0.315 | 0.129 | 0.958 | 0.278 |
| **Leon3** | 8 | 3.000 | 1.910 | 0.625 | 0.477 | 2.839 | -0.250 | 1.000 | 0.375 | 1.000 |
| **Leon11** | 9 | 3.000 | 2.418 | 0.889 | 0.586 | 2.925 | -0.471 | 1.000 | 0.071 | 0.274 |
| **Leon15** | 11 | 1.000 | 1.000 | 0.000 | 0.000 | 1.000 | - | - | - | - |
| **Leon21** | 7 | 3.000 | 1.815 | 0.429 | 0.449 | 2.868 | 0.122 | 0.413 | 0.829 | 0.435 |
| **Leon30** | 7 | 2.000 | 1.690 | 0.571 | 0.408 | 1.999 | -0.333 | 1.000 | 0.538 | 1.000 |
| **Leon31** | 11 | 1.000 | 1.000 | 0.000 | 0.000 | 1.000 | - | - | - | - |
| **Leon35** | 11 | 1.000 | 1.000 | 0.000 | 0.000 | 1.000 | - | - | - | - |
| **Mean** | **9** | **2.083** | **1.763** | **0.450** | **0.347** | **2.043** | **-0.243** | **0.979** | **0.025*** | **-** |
| **Captive Metapopulation** | | | | | | | | | | |
| **Lchu01** | 32 | 2.000 | 1.789 | 0.594 | 0.441 | 2.000 | -0.333 | 0.988 | 0.067 | 0.107 |
| **Lchu6** | 32 | 2.000 | 1.679 | 0.563 | 0.404 | 2.000 | -0.378 | 1.000 | 0.038* | 0.069 |
| **Lchu7** | 29 | 2.000 | 1.918 | 0.793 | 0.479 | 2.000 | -0.647 | 1.000 | 0.004* | 0.000 |
| **Lchu8** | 31 | 3.000 | 1.387 | 0.290 | 0.279 | 2.997 | -0.025 | 0.558 | 0.600 | 0.310 |
| **Leon2** | 31 | 2.000 | 1.903 | 0.710 | 0.475 | 2.000 | -0.483 | 1.000 | 0.013* | 0.010 |
| **Leon3** | 32 | 2.000 | 1.983 | 0.281 | 0.496 | 2.000 | 0.445 | 0.029 | 0.996 | 0.015 |
| **Leon11** | 32 | 2.000 | 1.998 | 0.969 | 0.500 | 2.000 | -0.938 | 1.000 | 0.004* | 0.000 |
| **Leon15** | 31 | 4.000 | 1.975 | 0.645 | 0.494 | 3.935 | -0.292 | 1.000 | 0.025* | 0.259 |
| **Leon21** | 32 | 2.000 | 1.983 | 0.906 | 0.496 | 2.000 | -0.824 | 1.000 | 0.004* | 0.000 |
| **Leon30** | 32 | 2.000 | 1.998 | 0.781 | 0.500 | 2.000 | -0.553 | 1.000 | 0.008* | 0.004 |
| **Leon31** | 30 | 2.000 | 1.946 | 0.700 | 0.486 | 2.000 | -0.426 | 1.000 | 0.013* | 0.026 |
| **Leon35** | 32 | 2.000 | 1.998 | 0.594 | 0.500 | 2.000 | -0.173 | 0.904 | 0.267 | 0.479 |
| **Mean** | **31** | **2.250** | **1.880** | **0.652** | **0.462** | **2.244** | **-0.397** | **1.000** | **0.004*** | **-** |

N: sample number; A_N_: number of alleles; A_E_: number of effective alleles; A_R_: allelic richness; H_O_: observed heterozygosity, H_E_: expected heterozygosity F_IS_: Inbreeding coefficient due to deviation from Hardy-Weinberg Equilibrium, P_DH_: p-values for the deficit of heterozygotes for the inbreeding coefficient F_IS_; P_EH_: p-values for the excess of heterozygotes for the inbreeding coefficient F_IS_, P_HWE_: p-value for the Hardy Weinberg equilibrium; *Statistically significant values.
