# Supplementary material for: Genetic monitoring in ex situ populations of the endangered primate Leontopithecus chrysopygus and integrative analyses with the wild founder population
Source: PLoS One. 2025 May 7;20(5):e0322817. doi: 10.1371/journal.pone.0322817 (PMC12057915; doi:10.1371/journal.pone.0322817)
Supplement: S4 Table — CPRJ: Primatology Center of Rio de Janeiro; FPZSP: Zoological Park Foundation of São Paulo. Metapopulation: captive metapopulation. MD: Morro do Diabo State Park AR: alleles richness, HE: expected heterozygosity, HO: observed heterozygosity, FIS: inbreeding coefficient. * Significant statistical differences P < 0.05. p-adj: Values after the Bonferroni correction. (DOCX) [file pone.0322817.s006.docx]

**S4 Table. Summary of the P-values for differences between the genetic diversity parameters of the captive and wild populations of *Leontopithecus chrysopygus.***

| **Populations** | | **F_IS_** | | **H_E_** | | **H_O_** | | **A_R_** | |
| --- | --- | --- | --- | --- | --- | --- | --- | --- | --- |
| **Group 1** | **Group 2** | **p-value** | **p.adj** | **p-value** | **p.adj** | **p-value** | **p.adj** | **p-value** | **p.adj** |
| CPRJ | DWCT | 0.93 | 1.00 | 0.43 | 1.00 | 0.82 | 1.00 | 0.06 | 0.44 |
| CPRJ | FPZSP | 0.62 | 1.00 | 0.93 | 1.00 | 0.40 | 1.00 | 0.28 | 0.94 |
| CPRJ | Metapop | 0.58 | 1.00 | 0.54 | 1.00 | 0.56 | 1.00 | 0.00* | 0.00* |
| CPRJ | MD | 0.35 | 1.00 | 0.64 | 1.00 | 0.07 | 0.72 | 0.23 | 0.94 |
| DWCT | FPZSP | 0.58 | 1.00 | 0.73 | 1.00 | 0.48 | 1.00 | 0.02 | 0.12 |
| DWCT | Metapop | 0.60 | 1.00 | 0.34 | 1.00 | 0.50 | 1.00 | 0.12 | 0.72 |
| DWCT | MD | 0.21 | 1.00 | 0.93 | 1.00 | 0.08 | 0.80 | 1.00 | 1.00 |
| FPZSP | Metapop | 0.98 | 1.00 | 0.82 | 1.00 | 0.75 | 1.00 | 0.00* | 0.00* |
| FPZSP | MD | 0.73 | 1.00 | 0.77 | 1.00 | 0.28 | 1.00 | 0.17 | 0.87 |
| Metapop | MD | 0.54 | 1.00 | 0.66 | 1.00 | 0.14 | 1.00 | 0.48 | 0.95 |

CPRJ: Primatology Center of Rio de Janeiro; FPZSP: Zoological Park Foundation of São Paulo. Metapopulation: captive metapopulation. MD: Morro do Diabo State Park A_R_: alleles richness, H_E_: expected heterozygosity, H_O_: observed heterozygosity, F_IS_: inbreeding coefficient.

* Significant statistical differences P < 0.05. p-adj: Values after the Bonferroni correction.
