# Supplementary material for: Genetic monitoring in ex situ populations of the endangered primate Leontopithecus chrysopygus and integrative analyses with the wild founder population
Source: PLoS One. 2025 May 7;20(5):e0322817. doi: 10.1371/journal.pone.0322817 (PMC12057915; doi:10.1371/journal.pone.0322817)
Supplement: S5 Table — (DOCX) [file pone.0322817.s007.docx]

**Table S5.** Summary of the effective population size (N_e_) confidence interval (Parametric and JackKnife), estimated with NeEstimator 2.0 software, for the Brazilian captive and wild populations of *Leontopithecus chrysopygus*.

| **Estimated population (N_e_)** | | | | | |
| --- | --- | --- | --- | --- | --- |
| **Population** |  | **Lowest Allele Frequency Used** | | | |
|  |  | **0.05** | **0.02** | **0.01** | **0+** |
| **FPZSP** | **Estimated Ne^ =** | Infinite | Infinite | Infinite | Infinite |
|  | **Parametric** | 28.2 | 25.1 | 25.1 | 25.1 |
|  |  | Infinite | Infinite | Infinite | Infinite |
|  | **JackKnife on Samples** | 12.8 | 17.5 | 17.5 | 17.5 |
|  |  | Infinite | Infinite | Infinite | Infinite |
| **CPRJ** | **Estimated Ne^ =** | 1.2 | 1.2 | 1.2 | 1.2 |
|  | **Parametric** | 0.6 | 0.6 | 0.6 | 0.6 |
|  |  | 2.5 | 2.5 | 2.5 | 2.5 |
|  | **JackKnife on Samples** | 0.5 | 0.5 | 0.5 | 0.5 |
|  |  | 3.2 | 3.2 | 3.2 | 3.2 |
| **DWCT** | **Estimated Ne^ =** | Infinite | Infinite | Infinite | Infinite |
|  | **Parametric** | Infinite | Infinite | Infinite | Infinite |
|  |  | Infinite | Infinite | Infinite | Infinite |
|  | **JackKnife on Samples** | 0.1 | 0.1 | 0.1 | 0.1 |
|  |  | Infinite | Infinite | Infinite | Infinite |
| **MD** | **Estimated Ne^ =** | 53.4 | 53.4 | 53.4 | 53.4 |
|  | **Parametric** | 1.6 | 1.6 | 1.6 | 1.6 |
|  |  | Infinite | Infinite | Infinite | Infinite |
|  | **JackKnife on Samples** | 1.8 | 1.8 | 1.8 | 1.8 |
|  |  | Infinite | Infinite | Infinite | Infinite |
| **Metapopulation** | **Estimated Ne^ =** | 10.1 | 11.9 | 12.6 | 12.6 |
|  | **Parametric** | 3.5 | 3.8 | 3.9 | 3.9 |
|  |  | 25.5 | 32.5 | 35.4 | 35.4 |
|  | **JackKnife on Samples** | 2.2 | 2.4 | 2.8 | 2.8 |
|  |  | 94.9 | 224.9 | 103.0 | 103.0 |

CPRJ: Primatology Center of Rio de Janeiro; FPZSP: Zoological Park Foundation of São Paulo; DWTC: Durrell Wild Conservation Trust; MD: Morro do Diabo State Park; Metapopulation: captive Metapopulation in 2020. Note: For captive groups, Ne values resulted in “infinity” or values below 10 (see Table S5). "Infinite" values ​​mean that evidence of genetic variation caused by a finite number of parents was not found (Do e tal., 2014)
